# Supplementary material for: Consistency of superb microvascular imaging and contrast-enhanced ultrasonography in detection of intraplaque neovascularization: A meta-analysis
Source: PLoS One. 2020 Jul 30;15(7):e0230937. doi: 10.1371/journal.pone.0230937 (PMC7392312; doi:10.1371/journal.pone.0230937)
Supplement: S1 Checklist — (DOC) [file pone.0230937.s001.doc]

| **Section/topic** | **#** | **Checklist item** | **Reported on page #** |
| --- | --- | --- | --- |
| **TITLE** | | |  |
| Title | 1 | Consistency of superb microvascular imaging and contrast enhanced ultrasonography in detecting intraplaque neovascularization: a meta-analysis | 1 |
| **ABSTRACT** | | |  |
| Structured summary | 2 | This meta-analysis aimed to identify the consistency of superb microvascular imaging(SMI) and contrast-enhanced ultrasonography(CEUS) in detecting intraplaque neovascularization(IPN). We searched PubMed, Web of Science, Cochrane Library, CISCOM, and CBM databases without language restrictions. Meta-analysis was conducted using STATA version 15.1 software. We calculated the pooled Kappa index. Ten studies that met all inclusion criteria were included in this meta-analysis. A total of 608 carotid plaques were assessed through both SMI and CEUS. The pooled summary Kappa index was 0.743(95 % CI=0.696-0.790) with statistical significance( z= 31.14, p<0.01). We found no evidence for publication bias (t=-1.21, p=0.261). Our meta-analysis indicates that SMI and CEUS display a good consistency in detecting IPN of carotid plaque, that is to say SMI ultrasound maybe a promising alternative to CEUS for detecting IPN of carotid plaque. | 2 |
| **INTRODUCTION** | | |  |
| Rationale | 3 | In today's society, atherosclerosis incidence rate is high, and tends to be younger. Atherosclerotic plaque can lead to carotid artery stenosis, affect the blood supply of carotid artery to the brain, and vulnerable carotid atherosclerotic plaques are prone to rupture and bleeding to form thrombus, which can enter the blood vessels of the brain with the blood, causing ischemic stroke events. Stroke is a common refractory disease that seriously endangers human health and life safety. The development of atherosclerotic plaque seriously affects the outcome and prognosis of the disease, and there is a significant consistency between IPN and atherosclerotic plaque vulnerability, so the IPN can be used as a high-risk factor to evaluate the vulnerability of plaque. CEUS can visualize IPN effectively, but is an invasive examination requiring injection of contrast medium. SMI is a new ultrasonic diagnosis technology which uses adaptive principle to display low-speed blood flow signal.Several studies had suggested that SMI , as a promising noninvasive alternative, can detect IPN with accuracy comparable to CEUS[7]. However, the results of these studies have been contradictory and the sample sizes were small. Therefore, we performed the present meta-analysis to identify the consistency of SMI and CEUS in detecting intraplaque IPN. | 2 |
| Objectives | 4 | Participants carotid plaque；interventions SMI；comparisons CEUS；outcomes IPN, and study design：clinical cohort study | 2 |
| **METHODS** | | |  |
| Protocol and registration | 5 | N/A. |  |
| Eligibility criteria | 6 | (1) the study design must be a clinical cohort study, (2) the study must relate to the comparison of CEUS and SMI for detecting IPN, (3) intraplaque microvascular flow (IMVF) were be graded, and (4)published data in the row x column tables must be sufficient for Kappa index and standard error.. If the study could not meet the inclusion criteria,it would be excluded. The most recent or the largest sample size publication was included when the authors published several studies using the same subjects. | 2 |
| Information sources | 7 | We searched PubMed, Web of Science, Cochrane Library, CISCOM, and CBM databases without language restrictions.We also performed a manual search to find other potential articles. | 2 |
| Search | 8 | **(((((Carotid[Title/Abstract]) AND ((((((((Plaques[Title/Abstract]) OR Plaque[Title/Abstract]) OR Fatty Streak, Arterial[Title/Abstract]) OR Arterial Fatty Streak[Title/Abstract]) OR Fibroatheroma[Title/Abstract]) OR Atheroma[Title/Abstract])) OR "Plaque, Atherosclerotic"[Mesh]))) AND (((Contrast[Title/Abstract]) AND (((Ultrasonic[Title/Abstract]) OR Ultrasound[Title/Abstract]) OR ultrasonography[Title/Abstract])) AND ((((vulnerability[Title/Abstract]) OR stability[Title/Abstract])) OR Neovascularization[Title/Abstract])))) AND superb microvascular imaging[Title/Abstract]**. | 2 |
| Study selection | 9 | Initially, the searched keywords identified 65 articles. We Reviewed the titles and abstracts of all articles and excluded 42 articles; full texts and data integrity were also reviewed and 10 were further excluded. Finally, 13 studies that met all inclusion criteria were included in this meta-analysis. | 3 |
| Data collection process | 10 | Relevant data were systematically extracted from all included studies by two researchers using a standardized form. | 3 |
| Data items | 11 | There searchers collected the following data: year of article, the first author’s surname, sample size, number of IMVF grades, number of every grade. | 3 |
| Risk of bias in individual studies | 12 | Methodological quality was independently assessed by two researchers according to a tool for the quality assessment of methodological index for non-randomized studies(MINORS) . | 3 |
| Summary measures | 13 | The MINORS criteria included 12 assessment items. Each of these items was scored as “yes” (2), “no” (0), or “unclear”(1). MINORS score ranged from 0 to 24; and score≥17 indicate a good quality. | 3 |
| Synthesis of results | 14 | The STATA version 15.1 software was used for Meta-analysis. We calculated the pooled summary OR and its 95% confidence interval(CI). The Cochran’s Q-statistic and I^2^ test were used to evaluate potential heterogeneity between studies. If Q test shows a P<0.05 or I^2^ test exhibits>50% which indicates significant heterogeneity, the random-effect model was conducted, or else the fixed-effects model was used. | 3 |

Page 1 of 2

| **Section/topic** | **#** | **Checklist item** | **Reported on page #** |
| --- | --- | --- | --- |
| Risk of bias across studies | 15 | We conducted Begger’s funnel plots and Egger’s linear regression test to investigate publication bias. | 3 |
| Additional analyses | 16 | In order to evaluate the influence of single study on the overall estimate, sensitivity analysis was performed. | 3 |
| **RESULTS** | | |  |
| Study selection | 17 | 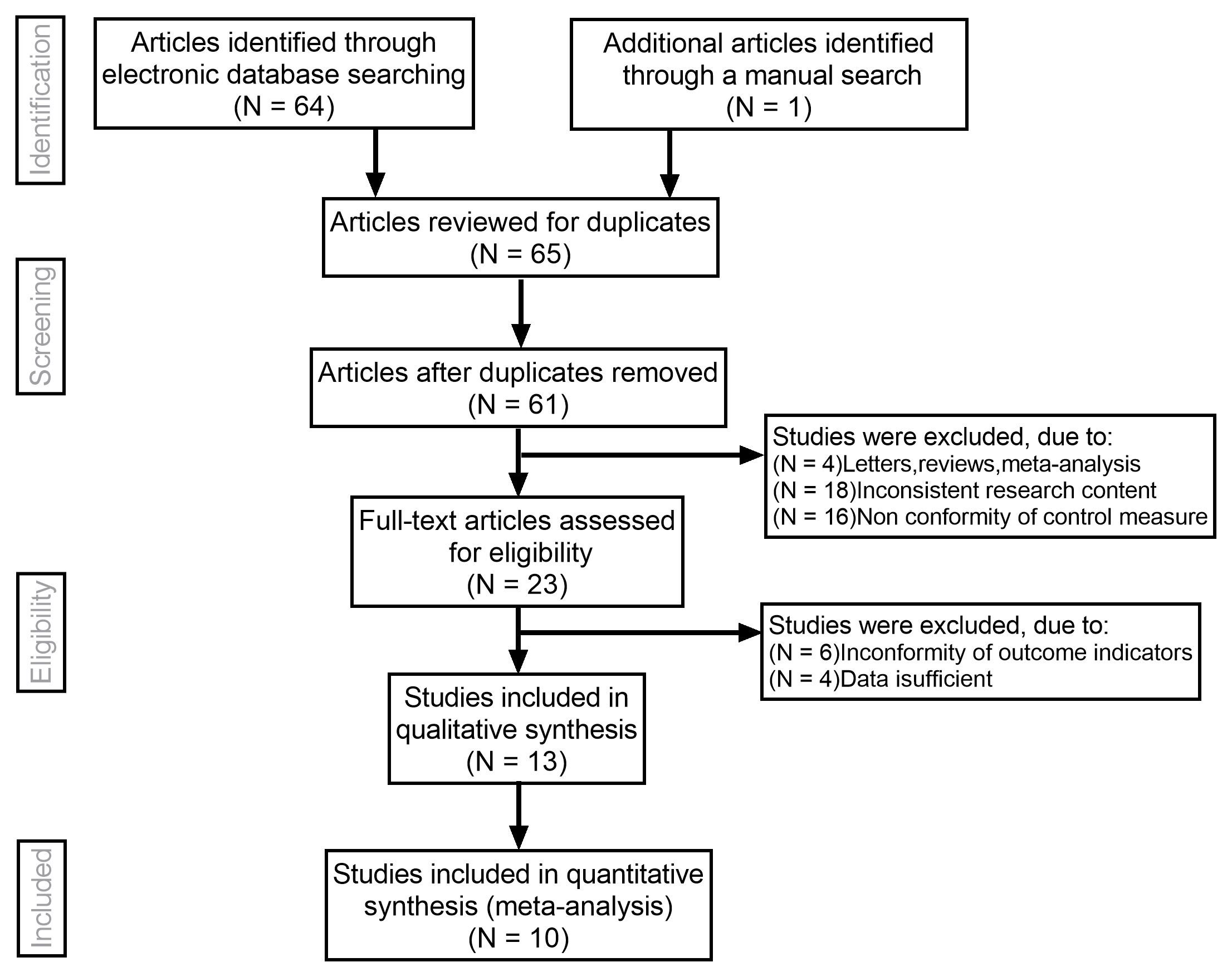 | 3 |
| Study characteristics | 18 | | Table1 Baseline characteristics and methodological quality of all included studies | | | | | | | | | | --- | --- | --- | --- | --- | --- | --- | --- | --- | | First author | Year | Sample size | Gender(M/F) | Age(years) | IMVF Grade number | Kappa index | SE(kappa) | MINORS score | | Ma L[10] | 2018 | 55 | 34/12 | 61±7 | 4 | 0.734 | 0.072 | 18 | | Chen JJ[11] | 2016 | 80 | 40/16 | 64.1±8.2 | 4 | 0.755 | 0.059 | 19 | | Zhang KY[12] | 2019 | 60 | 29/14 | 62.9±7.0 | 4 | 0.650 | 0.076 | 18 | | Ding Z[13] | 2019 | 62 | 50/12 | 61.59±9.16 | 3 | 0.769 | 0.068 | 18 | | Cheng LG[14] | 2015 | 57 | 44/13 | 61.8±7.8 | 3 | 0.607 | 0.127 | 19 | | Dong XY[15] | 2018 | 69 | 39/30 | 67.38±8.61 | 3 | 0.689 | 0.105 | 17 | | Zhang HX[16] | 2017 | 39 | 27/12 | 60±4 | 3 | 0.860 | 0.076 | 17 | | Ran HR[17] | 2018 | 33 | —— | —— | 4 | 0.621 | 0.103 | 19 | | Xie X[18] | 2018 | 108 | 53/16 | 68.1±8.8 | 4 | 0.748 | 0.054 | 20 | | Wang W[19] | 2019 | 45 | 33/12 | 61.42±7.37 | 4 | 0.839 | 0.090 | 18 | | M male, F female, IMVF intraplaque microvascular flow, MINORS methodological index for non-randomized studies, SE standard error | | | | | | | | | | 3 |
| Risk of bias within studies | 19 | | First author | Year | IMVF Grade number | MINORS score | | --- | --- | --- | --- | | Ma L | 2018 | 4 | 18 | | Chen JJ | 2016 | 4 | 19 | | Zhang KY | 2019 | 4 | 18 | | Ding Z | 2019 | 3 | 18 | | Cheng LG | 2015 | 3 | 19 | | Dong XY | 2018 | 3 | 17 | | Zhang HX | 2017 | 3 | 17 | | Ran HR | 2018 | 4 | 19 | | Xie X | 2018 | 4 | 20 | | Wang W | 2019 | 4 | 18 | | 3 |
| Results of individual studies | 20 | 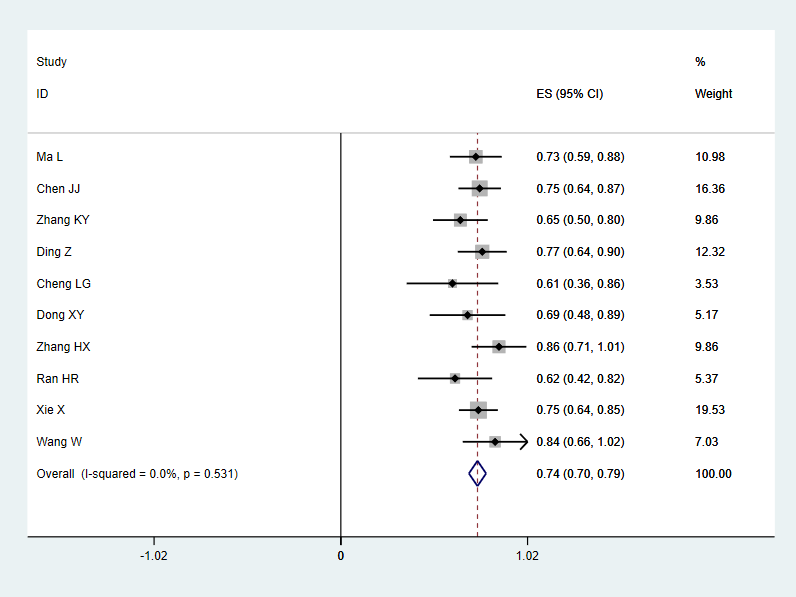 | 3 |
| Synthesis of results | 21 | The pooled summary Kappa index was 0.743(95 % CI=0.696-0.790) with statistical significance( z= 31.14, p<0.01) | 3 |
| Risk of bias across studies | 22 | Egger’s test also did not display strong statistical evidence for publication bias(t=1.21, p=0.261). | 3 |
| Additional analysis | 23 | Sensitivity analysis was carried out, and none of them caused obvious interference to the results. | 3 |
| **DISCUSSION** | | |  |
| Summary of evidence | 24 | SMI and CEUS display a good consistency in detecting IPN of carotid plaque。 | 3 |
| Limitations | 25 | Firstly,our results had lacked sufficient statistical power due to relatively small sample size and low-quality included studies. On the other hand, meta-analysis is a retrospective study that may lead to subject selection bias. Thirdly, this meta-analysis failed to obtain original data from the included studies, which may limit further clinical assessment of values of SMI in detecting IPN. Importantly, the majority of included studies originated from China, which may adversely affect the reliability and validity of our results. | 3 |
| Conclusions | 26 | SMI ultrasound is a promising alternative to CEUS for detecting IPN of carotid plaque. | 5 |
| **FUNDING** | | |  |
| Funding | 27 | N/A |  |

*From:*  Moher D, Liberati A, Tetzlaff J, Altman DG, The PRISMA Group (2009). Preferred Reporting Items for Systematic Reviews and Meta-Analyses: The PRISMA Statement. PLoS Med 6(7): e1000097. doi:10.1371/journal.pmed1000097

For more information, visit: **www.prisma-statement.org**.

Page 2 of 2
