# Supplementary material for: Consistency of superb microvascular imaging and contrast-enhanced ultrasonography in detection of intraplaque neovascularization: A meta-analysis
Source: PLoS One. 2020 Jul 30;15(7):e0230937. doi: 10.1371/journal.pone.0230937 (PMC7392312; doi:10.1371/journal.pone.0230937)
Supplement: Attachment — Submitted filename: Response to Reviewers.docx [file pone.0230937.s002.docx]

**Journal Requirements:**

1. Please ensure that your manuscript meets PLOS ONE's style requirements, including those for file naming.

OK.

2. We noticed you have some minor occurrence of overlapping text with the following previous publication(s), which needs to be addressed:

Appropriate modifications have been made.

3. PLOS requires an ORCID iD:

OK.

4. Please include your tables as part of your main manuscript and remove the individual files. Please note that supplementary tables (should remain/ be uploaded) as separate "supporting information" files.

OK.

5. Please include captions for your Supporting Information files at the end of your manuscript, and update any in-text citations to match accordingly.

OK.

**Reviewer #1:**

1) Did all studies use the same device from one manufacturer? What’s the potential impact on conclusion?

Yes, superb microvascular imaging(SMI; Toshiba Medical Systems Corporation, Tochigi, Japan).

All devices are from Toshiba aplio series color ultrasonic diagnostic instrument, which may reduce sources of heterogeneity.

2) Did the interrater-reliability studies include especially concerning about the grading?

No, the main statistical indicator is Kappa index. Risk of bias within studies was assessed through MINORS.

3) Is it possible to examine the plaque in the same plane comparing both methods?

Not always. Ultrasound usually scans in multiple sections, and takes the section with the most blood flow.

4) Authors should make appropriate changes to the language in standard English.

OK.

**Reviewer #2:**

**First**, all the included 10 literatures were published in Chinese journals. How do the authors control the publication bias if there is only one kind of language literatures? Actually, there may be some international literatures which would meet the inculsion criteria. For example if such papers were published in one language neighter in chinese nor in English, but met the inclusion criteria.

We have searched PubMed, Web of Science, Cochrane Library, and CBM databases in fact, but only the 10 literatures are eligible. The Begg’s funnel plot and Egger’s test were applied to assess the publication bias. We have add descriptionin ‘Although existing systematic reviews have suggested that the inclusion of articles published in Chinese alone would not affect the overall effect direction, the exclusion of publications in other languages may reduce the precision of the summary effect Estimates’ in the discussion part.

**Second**, in the 10 literatures, some of the results were given double-blind evaluation by different doctors, others were not, how about the rate of deviation and heterogeneity caused by such issue?

Empirical evidence suggests that lack of blinding tends to lead to overestimation of the treatment effect. This implies that even if bias is introduced by the lack of blinding, the true Kappa index would be even smaller than the results generated by this systematic review.

**Third**, in the discussion part, though the authors indicate the limitations of this meta-analysis, there are no detailed measures to salvage them. The authors should address the objective reasons of the questions, and give some suggestions for next work.

OK.

**In addition**, there are some inappropriate sentences and words in the manuscript. Such as, in the first sentence of the Introduction part: “In today’s society, ….to be younger” and in the seventh sentence of this paragraph: “Several studies had…comparable to CEUS”, and so on. It seemed the English expression was incorrect. Please make serious changes of the language by someone with expertise in technical English editing before resubmitting.

OK.
