# Supplementary material for: Consistency of superb microvascular imaging and contrast-enhanced ultrasonography in detection of intraplaque neovascularization: A meta-analysis
Source: PLoS One. 2020 Jul 30;15(7):e0230937. doi: 10.1371/journal.pone.0230937 (PMC7392312; doi:10.1371/journal.pone.0230937)
Supplement: Attachment — Submitted filename: Response to Reviewers.rtf [file pone.0230937.s003.rtf]

1.In the Discussion part about limitations some suggestions should be mentioned for next work.
Answer:OK.--line160-162.
2.Please make further careful polish of language.
Answer:OK.
3.While revising your submission, please upload your figure files to the Preflight Analysis and Conversion Engine (PACE) digital diagnostic tool.
Answer:OK.
